# My child is growing and now? Exploring the environmental needs of children with congenital Zika syndrome according to their caregivers' perceptions

**DOI:** 10.1111/hex.13587

**Published:** 2022-10-25

**Authors:** Monique L. G. Coelho, Taynah N. C. Campos, Adriana G. Magalhães, Jean B. Felix, Adriana Melo, Jousilene S. Tavares, Karolline S. Monteiro, Egmar Longo

**Affiliations:** ^1^ Faculty of Health Science of Trairi Federal University of Rio Grande do Norte Santa Cruz Brazil

**Keywords:** caregivers, children, congenital Zika syndrome, environment, participation

## Abstract

**Introduction:**

Promoting social inclusion of children with congenital Zika virus syndrome (CZS) is challenging, mostly, when there is a transport problem, low access to information and a long distance between the house and health services. Participation can be understood as involvement in a life situation and is strongly influenced by physical, social and attitudinal environmental factors; however, was still little explored in the case of children with CZS. In this sense, this study aimed to explore the perception of caregivers about the environmental needs of children with CZS, differentiating barriers and facilitators.

**Methods:**

This is qualitative research. Thematic analysis was used to identify the environmental needs perceived by caregivers of children with CZS. The patient public involvement (PPI) approach was incorporated with the purpose of validating the data analysis performed by the researchers. After this step, the data were categorized in terms of barriers and facilitators and validated by the group of researchers.

**Results:**

A relevant environmental need reported by caregivers as a barrier was social support for children with CZS. Ableism was also evidenced as an important attitudinal barrier. Health services were essential for the lives of children with CZS and the availability of auxiliary devices as facilitators of participation. Environmental factors related to medication and food routines were, for the most part, facilitators.

**Conclusion:**

This study contributes to critical approaches to the impacts linked to environmental factors of children with CZS, recognition of these children is an evolving process and fundamental to basic rights for adequate living in society. The data point to the need to implement public policies aimed at children with CZS, as well as the availability of qualified professionals to apply family‐centred care and skills‐focused management. Building friendly environments that promote broad social participation will contribute to the healthy growth of children with CZS.

**Patient or Public Contribution:**

Six caregivers (20% of the caregivers) as part of the PPI approach were contacted and participated in individual virtual meetings to discuss the results of the thematic analysis regarding the environmental needs of children with CZS.

## 1 INTRODUCTION

Disabilities affect between 93 and 150 million children worldwide and 3.5 million children in Brazil.^1^, ^2^ Among the Brazilian children with disabilities, 3563 were diagnosed with congenital Zika virus syndrome (CZS), and 19,492 other cases are suspected of CZS.^3^ CZS was described in Brazil after a microcephaly outbreak between 2015 and 2016. Most children with CZS have neuro psychomotor impairment (commonly associated with microcephaly), arthrogryposis, visual and intellectual deficits, epilepsy^4^ and speech‐language disorders.^5^

Promoting social inclusion of children with CZS is challenging and requires lifelong professional assistance, psychological, social support and family‐centred interventions.^6^, ^7^, ^8^ In this sense, the International Classification of Functioning, Disability and Health (ICF) defines participation as the ‘involvement in a life situation’ and it is influenced by environmental factors, such as physical, social and attitudinal aspects (i.e., physical features of the house and school, relationship to close family, community activities, health services, communication and transportation).^9^, ^10^

Children with disabilities have restricted access to health and educational services compared with children without disabilities.^11^, ^12^ However, monitoring children with developmental disabilities is demanding and possibly not equitable. In Brazil, children with CZS have the right to diagnosis, childcare, early intervention and integral and continuous specialized attention in the Unified Health System (SUS).^13^, ^14^ However, the offer of these services is still insufficient and fragmented, and a lack of communication for orienting and providing social support to children and their families is observed between different levels of the health system. Furthermore, mothers and health workers disclose flaws in access to organizational, sociocultural, geographic and economic services.^15^, ^16^

The difficulties faced by many caregivers are related to socioeconomic factors, such as expenditure on therapies and food, in addition to low family and community support.^17^ Family and community support are pointed out as essential in facing the difficulties of caregivers of children with CZS. It is also known that caregivers of children with CZS tend to be more likely to develop mental health problems that worsen over time, mainly related to self‐perception of health, depression and anxiety problems.^18^

Caregivers of children with CZS are mostly their mothers, who have low educational and socioeconomic levels, no formal work and were abandoned by their partners.^16^, ^19^ Lack of knowledge and funding for transportation, low access to information, long distance between the house and health services, need for a private health plan to access specific treatments, work instability and fear of dismissal hamper the access to health services.^19^ In addition, the uncertainty about the development and future of children with CZS overwhelms mothers.^7^ Therefore, mapping the environmental needs of children with CZS may facilitate social inclusion and participation over time.

As with other developmental disabilities, children with CZS are at a disadvantage in terms of full participation in society.^20^ Studies that analysed the social and developmental context of children with disabilities highlight the importance of participation as a positive outcome of rehabilitation and health for these children. However, the physical, social and attitudinal environments, despite being potentially modifiable and determinant for the results of participation of children with disabilities, were still little explored in the case of children with CZS. Most children with disabilities still face many environmental barriers, especially those living in low‐income countries.^21^, ^22^

Two previous research briefly investigated access to health services and social support of mothers and health professionals who took care of children with CZS.^15^, ^16^ However, both studies did not deeply explore all relevant aspects of the environment, such as the use of products and technologies and the behaviours and relationships of family, friends and healthcare professionals. In this sense, this study aimed to explore the perception of caregivers about the environmental needs of children with CZS, differentiating barriers and facilitators.

## 2 METHODS

This is a qualitative research study that involved children with CZS and their caregivers in three rehabilitation centres. This study was carried out in Santa Cruz, a small municipality in the State of Rio Grande do Norte, Northeast and Brazil, where the majority of the population is considered to be of low sociodemographic level. About 67% of CZS reported between 2015 and 2016 were in the Brazilian Northeast. Brazil's prevalence of CZS confirmed was 3.8/10 and 3.1/10 thousand live births in 2015 and 2016, respectively, while in Northeast Brazil it was 12.6 and 7.1/10 thousand live births in the same years. Therefore, the data suggest that CZS is associated with a complex combination of biological, environmental and socioeconomic factors.^23^, ^24^, ^25^

### 2.1 Main scope of the study

#### 2.1.1 Recruitment and sampling

Thirty‐two caregivers (mothers or grandmothers or both) were selected using intentional sampling. Data were collected in person and using phone calls. Caregivers of 32 children with CZS diagnosed between 0 and 5 years old and users of rehabilitation services within the area of study were included.

Caregivers received permanent information about study aims and procedures and provided written consent. This study followed the Declaration of Helsinki and was approved by the research ethics committee of the Federal University of Rio Grande do Norte (no. 2.357.552) and the University of Liverpool (no. 2083).

#### 2.1.2 Data collection

Semi‐structured interviews with a participative approach were performed through focal groups of 4–6 caregivers and duration between 60 and 90 min (until data saturation). Questions followed an adapted questionnaire based on the ICF, and are detailed in a previous study.^26^ Researchers selected only questions related to environmental factors to understand the needs of children with CZS from the perspective of caregivers. Data of children with CZS regarding socioeconomic, mobility, rehabilitation, frequency in daycare or schools, epilepsy and vision, hearing or sleep disorders were also collected. All interviews were recorded, transcribed and thematically analysed.

### 2.2 Data analysis

Thematic analysis of qualitative data was used to identify the environmental needs perceived by caregivers of children with CZS,^27^ using the NVivo® software. The stages of thematic analysis were as follows: searching themes, reviewing and naming themes and producing the report.

**Figure 1:**
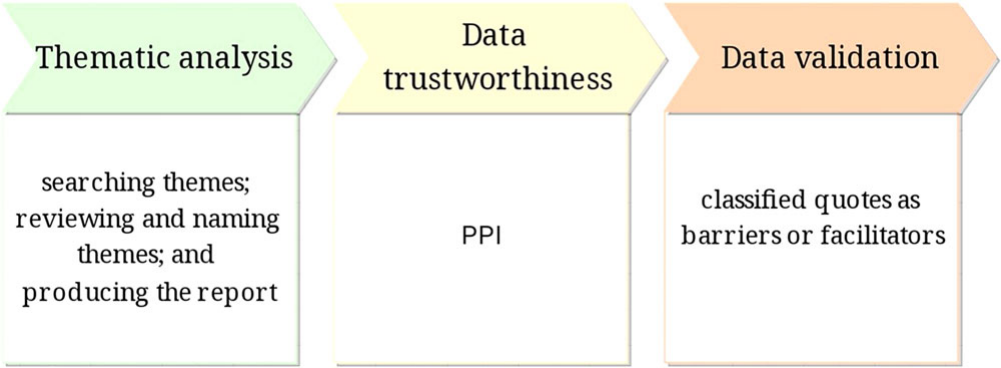
Methodology workflow. PPI, public and patient involvement.

Using a public and patient involvement (PPI) approach, researchers and caregivers reviewed the qualitative data in a stage called ‘data trustworthiness’. Data were classified as barriers or facilitators and were further validated by members of the research team. The methodology workflow is presented in Figure 1.

#### 2.2.1 Thematic analysis

The thematic analysis developed the general theme, whose meaning was derived from transcripts. This study focused on environmental factors defined in the ICF. Repetition of expressions throughout transcripts characterized groups of information that were organized in representative themes regarding the reality of children with CZS.

##### Searching themes

Transcripted phrases and fragments (i.e., quotes) were used for data exploration. Data were classified into main themes according to relevance during focal groups.

##### Reviewing and defining and naming themes

Codes were assigned to quotes. Three researchers with experience in qualitative studies with children with disabilities defined themes for each quote group.

##### Producing the report

Quotes of caregivers were grouped in specific themes and resulted in a map of themes. Quotes were categorized following the ICF, grouping environmental factors as qualifying constructs (barriers or facilitators) according to the context of children with CZS.

#### 2.2.2 Data trustworthiness—PPI

Ten themes derived from the thematic analysis were submitted for collaborative review by six caregivers (20% of the sample size) as part of the PPI approach.^28^ Participants were contacted and participated in individual virtual meetings to discuss the results of the thematic analysis regarding the environmental needs of children with CZS. Themes and quotes transcribed during data collection were schematized and randomly displayed to the selected caregivers. They weighed the correspondence of themes and quotes, generating a new thematic scheme.

#### 2.2.3 Data validation

The first author classified quotes as barriers or facilitators for data validation using ICF qualifiers. Two authors independently analysed data categorization. This analysis resulted in a concept map.

## 3 RESULTS

Sociodemographic analysis indicated that most participants belonged to low‐income households (i.e., earn less than a minimum wage). Some families earned between two and three minimum wages, and only one family earned higher than five minimum wages. Most caretakers had elementary or high school education. Only seven reached tertiary education (Table 1).

**Table 1:** Sociodemographic characteristics of participants

Variables	*n*	%
Caregivers' education		
Elementary school	10	31.2
High school	15	46.9
University education	07	21.9
Family monthly income (minimum wage*)		
Up to 1	17	53.1
Between 2–3	11	34.4
Between 3–4	1	3.1
No information	3	9.4
Age of children with CZS (years)		
Up to 02	02	6.2
Between 02–03	18	56.3
Between 03–04	12	37.5
City of residence/state		
Santa Cruz/RN	05	15.6
João Pessoa/PB	06	18.7
Campina Grande/PB	21	65.7

* Currently, the Brazilian minimum wage per month is BRL $1,212 ($ 242, 4 dollars).

Environmental needs identified in quotes of caregivers were organized in the following 10 main themes: family relationships; social support; community participation; public health policy; delivery of health services; physical and rehabilitation medicine; accessibility; medication adherence; staple food; auxiliary devices for disabled people. Those themes were denominated ‘first result’ and revealed the perception of caregivers of children with CZS.

**Figure 2:**
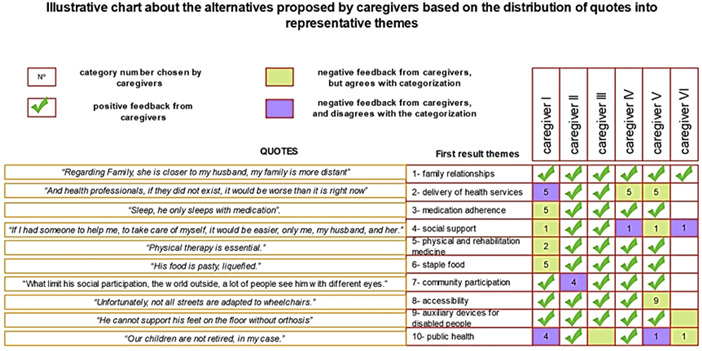
PPI scheme on the first result. PPI, public and patient involvement.

A PPI‐based process tested data *trustworthiness* (denominated ‘second result’). Feedback from caregivers demonstrated similarities between the first and second results. Representative phrases in each semantic group were similar to one of the ten themes from thematic analysis, as shown in Figure 2.

However, caregivers modified some classifications of representative phrases. Four out of six caregivers indicated similarities between ‘social support’ and ‘family relationships’, and three reported ‘public policies’ as equivalent to ‘social support’. Furthermore, three out of six caregivers understood that the phrase attributed to ‘delivery of health services’ was related to ‘physical and rehabilitation medicine’. Therefore, the second result combined themes and a newly organized nomenclature according to insights of caregivers. Seven thematic groups and their respective quantitative quotes (q) were characterized: social support (27q); access to health service (61q); community participation (05q); staple food (16q); spatial accessibility (19q); medication adherence (14q) and assistive technology (30q).

**Figure 3:**
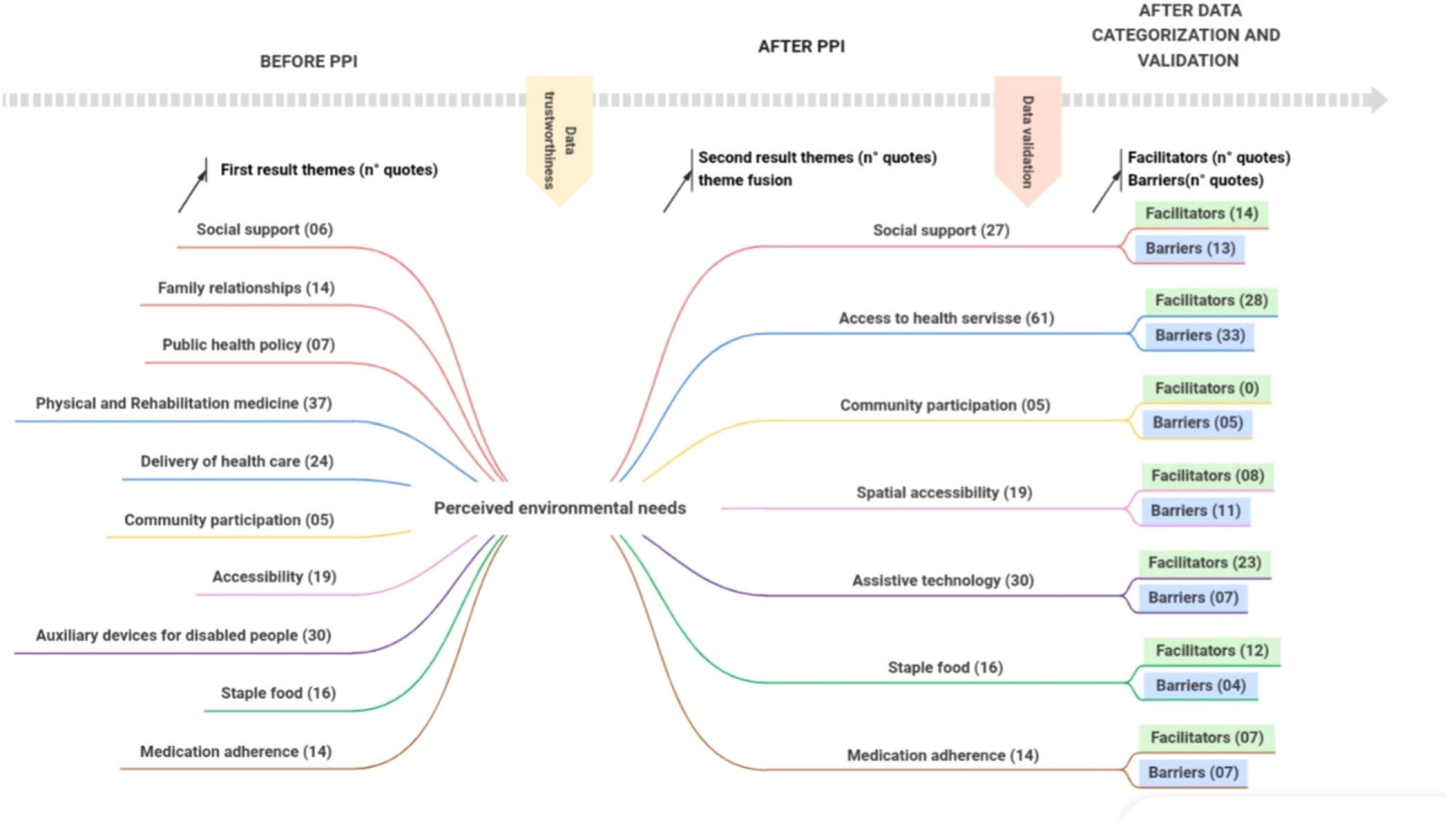
Distribution of quotes by themes according to methodological step. PPI, public and patient involvement.

After PPI, the researchers divided the quotes of the seven themes into barriers (B) or facilitators (F), shown quantitatively as (B)/(F): social support 13(B)/14(F); access to health service 33(B)/28(F); community participation 5(B)/0(F); staple food 4(B)/12(F); spatial accessibility 11(B)/8(F); medication adherence 7(B)/7(F) and assistive technology 7 (B)/23(F). The concept map summarizing thematic analysis (covering the results of the methodological steps and considering barriers and facilitators) is shown in Figure 3.

### 3.1 Themes

Themes related to the need for implementation of support policies and difficulties in inclusion and social recognition of children with CZS are represented in quotes. Health services access was essential for physical and cognitive development, and sociodemographic factors were equally important since they affect treatment adherence. Some quotes showed the importance of adequate spatial structure (e.g., residences, public spaces and public transportation) and strategies for dietary and medication routines. Representative quotes about the environmental needs of children with CZS are shown in Table 2.

**Table 2:** Quotes grouped according to barriers and facilitators

Theme	Perceived environmental needs	B	F
	Quotes		
Social support	‘About family relationships, she has difficulties identifying the family…’ (A)	√	
	‘And the prejudice in family is an obstacle for me…’ (B)	√	
	‘The government should help. Because we should not be wasting time…’ (C)	√	
	‘She has a great relationship with her family…’ (D)		√
	‘He loves his family, if it was possible, he would live with his grandparents, he loves his grandparents…’ (E)		√
	‘About family relationship, he recognizes all of them…’ (F)		√
Community participation	‘Everyone see her as normal, only when we get outside people look and ask absurd questions…’ (G)	√	
	‘The greatest difficulty is the prejudice…’ (H)	√	
Access to health service	‘Health services are precarious, but he still has it…’ (I)	√	
	‘I guess that many leave their child at the hospital and health professional does not know how to take care, so we need to instruct them…’ (J)	√	
	‘After “PediaSuit” he became another person’ (C)		√
Spacial accessibility	‘If you live in a not structured city, like one adapted to a wheelchair, the child will be without judgment (here is a slang that means intellectual disability), because rocks a lot…’ (L)	√	
	‘The greatest difficulty is transportation…’ (M)	√	
	‘Mobility products would facilitate a lot. We need a car, a wheelchair…’ (N)	√	
	‘Technology products for mobility or transportation are essential for him…’ (B)	√	
Assistive technology	‘Many children do not have access to wheelchair…’ (D)	√	
	‘After she started using her glasses, she is more active…’ (R)		√
	‘He only wants to give little steps if you put his orthosis on his feet…’ (E)		√
	‘The glasses are what helps hers to participate in everything…’ (G)		√
Medication adherence	‘The medication is expensive, we bought…’ (M)	√	
	‘I would like Science to produce medication for this disease’		√
	‘He takes medication to control his seizures…’ (P)		√
	‘The medication helps a lot…’ (Q)		√
	‘“Cannabis” helps to improve his seizures…’ (O)		√
Staple food	‘We always need to leave food prepared…’ (H)	√	
	‘Her food is pasty, when it isn't pasty it's in pieces…’ (E)		√
	‘A canned of fiber, like a fiber and some medicine…’ (H)		√
	She improved her eating, she used to eat worst, Now, with ‘Canabidiol’ (O)		√

#### 3.1.1 Social support and community participation

Quotes related to ‘family support’ demonstrated positive aspects regarding the involvement of first‐degree relatives. Furthermore, quotes showed that children with CZS were able to recognize their relatives. On the other hand, the difficulties in facing everyday challenges and the lack of social support for families of children with CZS were identified as failures related to the implementation of effective public policies aimed at families of people with disabilities. Thus, lack of social support was a limiting factor for children with CZS and pronounced in cases of denial and ableism of family members.

The ‘community participation’ theme, an important aspect of health and well‐being, showed the need to recognize children with CZS as part of the community. All caregivers demonstrated an overview of ableism with children with deficiency as an attitudinal barrier to community participation.

#### 3.1.2 Access to health service

The theme ‘access to health service’ addressed access to general health and specialized rehabilitation services, essential for the development of children with CZS. The lack of qualified health professionals in primary health attention was pointed out as a barrier. Furthermore, rehabilitation treatments offered in public health systems were often spread in different rehabilitation centres, hindering access.

Health services were essential for children with CZS, and the offer of teaching‐service‐community (partnerships between universities and health services) in school clinics, composed of multiprofessional teams to improve the treatment of children with CZS, were participative spaces and important for knowledge construction. Although specialized resources were not offered in all rehabilitation centres, they were considered facilitators for the rehabilitation of children with CZS. Specialized methods (e.g., Padovan®, Bobath® and PediaSuit®) were also considered essential by caregivers.

#### 3.1.3 Spatial accessibility and assistive technology for people with disabilities

Spatial accessibility and assistive technology for people with disabilities may facilitate daily life and positively impact the social participation and autonomy of children with CSZ. Transportations, streets, parks, residences and assistive technology planned and adapted for people with disabilities were considered facilitators that enhanced the independence of children with CZS.

Reports highlighted that assistive technologies for people with disabilities (e.g., glasses, foot orthosis and wheelchair) were facilitators. However, difficulties in acquiring a wheelchair and lack of an urbanization plan for people with disabilities were barriers, restricting the autonomy of families and social participation. Lack of adapted transport and streets designed for the use of wheelchairs also configured factors that restricted the social participation of children with CZS. The means of transport were often restricted to cars and motorcycles.

#### 3.1.4 Medication adherence and staple food

Medication and dietary routine showed a positive impact on essential and adaptive factors in children with CZS. Reports of caregivers demonstrated the importance of medication and other therapeutic alternatives (e.g., cannabidiol) for health stability and to enhance the quality of life of children with CZS.

Caregivers also described different adaptations in the dietary routine of children with CZS. Pasty food and the use of thickeners and tubes for feeding children with severe deficits were considered facilitators. Furthermore, cannabidiol was associated with enhanced appetite and better digestion.

Caregivers reported medication access (high cost and flaws in public policies), adverse effects of drugs, lack of scientific protocols for treating CZS and restricted food elaboration as barriers.

## 4 DISCUSSION

This study contributed to the body of knowledge regarding environmental factors perceived by caregivers of children with CZS in Brazil. Importantly, the study incorporated PPI as a collaborative strategy for knowledge development and recognition of caregivers' perspectives. The involvement of caregivers in the research allowed access to aspects of the environmental needs of children with CZS and can lead to the implementation of strategies focused on reducing barriers and favouring friendly environments.^29^

Low family income is a barrier to the social participation of children with CZS, as observed in a previous study.^30^ As observed, most of the participants in our study belonged to low‐income families, with low education and all caregivers were female (mothers or grandparents), which reinforces a cycle of overload in women, already documented in other studies.^31^, ^32^ Encouraging recreational activities in spaces free of barriers in areas within the community can facilitate access and reduce this situation of social disadvantage observed through the statements of caregivers from our study. Disability and poverty are believed to operate in a cycle, each reinforcing the other^33^ and our results appear to reinforce these findings. Poverty is a factor that negatively affects the level of empowerment of families and access to rehabilitation treatments.^8^, ^30^ Public policies that promote income and equal opportunities can help to minimize this gap between families of children with disabilities when compared to their peers without disabilities. Our findings reinforce the need for support through public policies, especially for a population of low‐income caregivers.

A comparative study demonstrated that children with disabilities from low‐income families had disadvantages regarding social and community participation, access to health service and self‐care compared with peers from high‐income families.^10^ Moreover, those results corroborated with our caregivers' complaints about the socioeconomic scenario, evidencing the need for complex dynamics for the care of children with CZS. According to the caregivers, the public health services offered therapeutic support. However, the rehabilitation services were delivered in different centres (even in other cities), characterizing a fragmented flow of care. Although all were assisted by the SUS, rehabilitation services were not accessible to all families with children with CZS. Similar results were found in a recent study, where mothers of children born with microcephaly faced barriers that prevented them from accessing the specialized health care needed for their children, as well as adequate psychosocial support for themselves.^17^

Rehabilitation for children with CZS was initially guided by the Ministry of Health guidelines, through recommendations for early intervention, from 0 to 3 years. Currently, as most children with CZS are between 5 and 6 years old, health care may be guided by the Policy of the National Plan for the Rights of Persons with Disabilities or the Living Without Limits Plan established in 2011 in Brazil.^34^ Recent findings on the perception of parents and health professionals about the care of children with CZS showed positive aspects of patient‐centred care, highlighting the quality of the SUS to meet the biopsychosocial demands of these children.^35^

Early access to health care has positive effects on children with CZS.^10^ Our results also indicated that adequate access to health services and spatial accessibility in cities is essential for the routine organization of families with children with CZS.^34^ The favourable disposition of health service (i.e., localization, spatial accessibility and transportation adequacy) for people with disabilities were a requirement for life planning of families, as observed in findings regarding the transition to adulthood of children with disabilities.^35^ Thus, those results showed the urgency of providing routine facilitators for adequate participation of people with disabilities in society.

Flaws in the implementation of fundamental rights and public policies aimed at the needs of minorities can impair the social support necessary for equity. The social support includes, but is not limited to, physical access to community environments and access to assistive technology devices. According to our caregivers, the inefficiency of political, social and structural support and ableism harmed the participation and recognition of children with CZS in society. These aspects are also observed in children with disabilities from low‐to‐middle income countries.^36^

Another finding highlighted the difficulty in accessing specialized rehabilitation intervention for children with CZS, such as the Padovan®, Bobath® and Pediasuit® methods. Caregivers who had access to intensive intervention with the use of therapeutic suits reported improvements in their children. Implementation of rehabilitation intervention for children with CZS should consider the principles of evidence‐based practice with long‐term benefits and a focus on improving participation.^37^ High‐cost interventions that have insufficient scientific evidence should be viewed with caution, especially for their implementation in public rehabilitation policies

Access to diet and medication, as strategies to improve adherence to the diet (cannabidiol, thickeners and tubes) were in line with results from previous studies.^38^ Although alternatives for helping or maintaining basic daily living activities (e.g., use of anticonvulsant drugs) may be obstacles in routine, such strategies were categorized as facilitators for social participation of children with CZS. Therefore, approaches focused on abilities rather than disabilities could increase the inclusion and encourage care focused on positive factors and capabilities of children with disabilities.^39^

The use of PPI, a collaborative approach to qualitative data, was the strength of this study. In addition, knowing the needs of children at a stage of growth, when environmental demands may be different from those of the first years of life, may support more effective interventions focusing on participation as an outcome.

However, the present research had some limitations. Only part of the caregivers was involved in the process of validating the qualitative analyses since most were not available. Furthermore, compensation was a limiting factor in strategies for public involvement in all phases of the study.^40^ In Brazil, the ethics committee forbids payment of participants in scientific studies, although caregivers dedicated time and provided experiences to construct solutions in health for children with CZS.

## 5 CONCLUSION

This study contributed to critical approaches to the impacts of environmental factors in children with CZS using a collaborative methodology that involves caregivers in research. Recognizing the needs of children growing up with CZS is a fundamental process to ensure the inclusion of these children in the community.

Children with CZS (and with other developmental disabilities) encounter barriers to social participation. Future studies should explore the school needs of children with CZS and the challenges related to inclusion. Effective public policies and efforts of health professionals should be centred on the family and promote an inclusive environment for adequate participation of children with CZS.

## AUTHOR CONTRIBUTIONS

Monique Coelho was involved in the data collection, analysis and interpretation of results, draft manuscript preparation and revision. Adriana Melo and Jousilene Tavares were involved in the study conception and data collection. Jean Felix was involved in the analysis and interpretation of results. Egmar Longo and Adriana Magalhães were involved in project supervision, study design, analysis, interpretation of results and manuscript review. Karoline Monteiro was involved in the analysis and interpretation of results, manuscript review and editing. All authors reviewed the results and approved the final version of the manuscript.

## CONFLICT OF INTEREST

The authors declare no conflicts of interest.

## Data Availability

Data can be made available, if necessary.
